# Accidental organophosphate insecticide intoxication in children: a reminder

**DOI:** 10.1186/1865-1380-4-32

**Published:** 2011-06-15

**Authors:** Willemijn van Heel, Said Hachimi-Idrissi

**Affiliations:** 1 Universitair Ziekenhuis Brussel (UZ Brussel), Paediatric Intensive Care Unit, Laarbeeklaan 101, 1090 Brussels, Belgium

**Keywords:** Plasma pseudocolinesterase, insecticides, intoxication, organophosphorus compound, antidote, children

## Abstract

Misuse of organophosphate insecticides, even in case of domestic application, can be life threatening. We report the case of siblings admitted with respiratory distress, pinpoint pupils and slurred speech. The symptoms appear after spraying the skin by insecticides. Plasma pseudocholinesterase level appeared to be very low, consistent with acute intoxication with organophosphate insecticide.

Management of organophosphate poisoning consists of airway management, administration of oxygen and fluid, as well as atropine in increasing doses and pralidoxime. Decontamination of the patient's skin and the removal of the patient's clothes are mandatory in order to avoid recontamination of the patient as well as the surrounding healthcare personnel.

Plasma pseudocholinesterase analysis is a cheap and an easy indicator for organophosphate insecticides intoxications and could be used for diagnosis and treatment monitoring.

## Introduction

Organophosphate insecticides are widely used in rural areas. Intentional ingestion of organophosphates is associated with a high mortality rate [1]. Organophosphate intoxication (OI) induces irreversible inhibition of acetylcholinesterase. Organophosphates phosphorylate the serine hydroxyl group of acetylcholine, leading to accumulation of acetylcholine at the cholinergic synapses [2]. This accumulation leads to weakness and fasciculation of the muscle. In the central nervous system, neural transmission is disrupted. If this blockade is not reversed within 24 h, large amounts of acetylcholinesterase are permanently destroyed [3].

Acetylcholinesterase is found in red blood cells as well as in nicotinic and muscarinic receptors. To determine the severity and/or the elimination time of OI, one should measure cholinesterase in blood, either by measuring plasma pseudocholinesterase (PCE) or by measuring the cholinesterase in erythrocytes (which is thought to reflect the cholinesterase in neurons and neuromuscular junctions). The first method is widely available and therefore commonly used [3,4].

Herein, we report a case of siblings who, upon being sprayed with an organophosphate solution, developed severe OI associated with central nervous system (CNS) depression.

## Case report

A 7-year-old previously healthy boy was brought into the emergency department with vomiting and reduced consciousness by his mother. He had been in good health until he was found, 30 min prior to admission, unresponsive in the bathroom. The mother was not able to provide more information.

At admission, the health care personnel had smelled an unspecified and unpleasant odour. The physical examination of the boy showed pinpoint pupils (2 mm diameter), hypersalivation and lacrimation. He was responsive to pain, but had slurred speech. His Glasgow Coma Scale (GCS) score was 9. Upon presentation, his vital signs included a rectal temperature of 36.8°C; heart rate, 117 beats/min; respiratory rate, 38 breaths/min; blood pressure, 112/58 mmHg; and haemoglobin saturation, 96%. Lung auscultation revealed bilateral wheezing. He had no abdominal tenderness, distension or hepatomegaly. The skin was warm and clammy with capillary refill (CR) of less than 2 s. Eight minutes after admission, his heart rate suddenly dropped down to 50 beats/min, followed by respiratory arrest. After orotracheal intubation, mechanical ventilation and atropine administration (0.02 mg/kg every 5 min), the patient's condition stabilized.

The cause of the symptoms was unclear, but intoxication with opiates or an organophosphorus compound (OC) was considered [5]. The patient's symptoms, the recovery after atropine administration and the occurrence of headache in the involved health care personnel indicated probable OI.

Shortly thereafter, the boy's 10-year-old sister, with the exact same unpleasant odour, altered sensorium, vomiting and respiratory distress, was brought to the emergency department by the father. She was afebrile and had a heart rate of 133 beats/min; the respiration was shallow at a rate of 31 breaths/min with bilateral wheezing and bronchial secretions. Her blood pressure was 131/76 mmHg, and the GCS was 15. Her pupils were 1 mm in diameter, and the CR was prolonged up to 4 s. She was stabilized with oxygen administration through a non-rebreathing mask and a 20 ml/kg bolus of saline fluid through a secured intravenous vascular catheter. Because OI had been suspected earlier for her brother, atropine (0.05 mg/kg) was given to prevent further decline.

Both children were transferred to the paediatric intensive care unit (PICU).

All laboratory values were normal, except for a decreased PCE. The boy's PCE was 0.3 kU/l and the girl's 0.2 kU/l (laboratory reference range: 4.6-10.4 kU/l).

These clinical and biological findings confirmed our diagnosis of OI.

Subsequently, the girl told us that they had been spraying fluid from a bottle while playing in the bathroom. Later on, the mother admitted that she had filled the bottle with pesticide to eradicate insects in the house, and subsequently analysis of the bottle's solution showed a high concentration of OC.

The boy was kept on mechanical ventilation for the next 24 h. He was treated with large fluid infusions, atropine (0.05 mg/kg every 15 min) and pralidoxime (25 mg/kg every 6 h).

**Figure 1:**
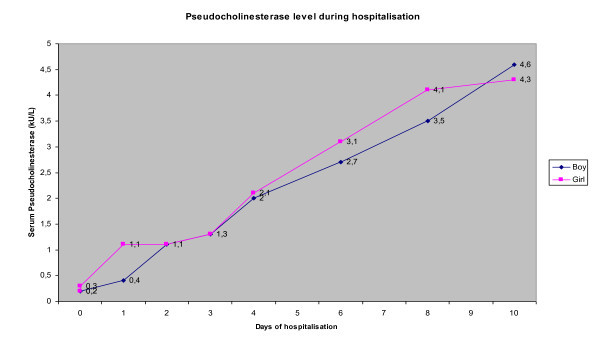
**Pseudocholinesterase levels of our patients during hospitalisation**.

The frequency of atropine administration was reduced and finally stopped when symptoms such as bradycardia, hypersecretion and bronchospams disappeared. Both patients improved considerably, although the boy showed fasciculations for an additional day. After the atropine treatment had been stopped, pralidoxime was slowly decreased and stopped after 6 days. His PCE level was 4.3 kU/l on day 10 (Figure 1).

The sister was treated with two doses of atropine (0.05 mg/kg) and pralidoxime (25 mg/kg every 6 h). The pralidoxime dosage was rapidly reduced and finally stopped after 4 days. Her PCE level was 4.6 kU/l on day 10 as well (Figure 1).

The children were discharged from the PICU on day 6 and from the hospital on day 10 without any sequelae. Further evaluation of the siblings 2 weeks later showed normal clinical findings, and the PCE values were within the normal range.

## Discussion

The striking similarity and timely fashion of the clinical presentation of these siblings suggested either a toxic environmental exposure or ingestion. Both children had some elements of CNS depression, respiratory difficulty, hypersecretion and miotic pupils. This constellation of findings is highly suggestive of a cholinergic toxidrome, and additional inquiry revealed exposure to OC.

OCs are commonly used in agricultural products, including insecticides and defoliants. They are rapidly absorbed by all routes of exposure, including dermal, respiratory and gastrointestinal, and irreversibly inhibit the enzyme acetylcholinesterase at cholinergic synapses, resulting in excess cholinergic stimulation at the neuromuscular junction, the sympathetic and parasympathetic nervous systems, and the CNS [3].

In our patients the absorption was probably via different routes, the skin, and the mouth, and/or via the respiratory tract while they were spraying the solution at each other in the bathroom.

The initial management should be directed toward securing and maintaining a stable patent airway and assuring adequate gas exchange and end-organ perfusion. Once these elements are stable and secure, efforts can be directed toward establishing a definitive diagnosis and treatment.

Unlike adults, infants mainly present with acute CNS depression [6] and do not demonstrate the typical muscarinic effects. Symptoms such as fasciculation, bradycardia and acute respiratory failure are more common in children [7].

Tachycardia, rather than bradycardia, has been noted upon presentation in 49% of children presenting with OI [6].

The acute respiratory failure in our cases was likely multifactorial in origin, resulting from secretions and bronchospasm from muscarinic stimulation. In addition, stimulation of nicotinic receptors causes weakness and paresis of the respiratory muscles [8].

The bradycardia event in our first case was most probably secondary to an apneic episode.

Acute OI is a clinical diagnosis. Red blood cell cholinesterase levels are usually markedly diminished, but this laboratory test is seldom readily available. Although plasma PCE levels may be diminished as well, still there is little correlation with acetylcholinesterase activity in either the brain or at the neuromuscular junction [4,9]. However, the decrease in PCE levels may serve as a marker of exposure to OC and supports the diagnosis. The diagnosis is therefore based on a history of exposure, recognition of the cholinergic toxidrome, and improvement or resolution of symptoms after appropriate treatment [4,9,10].

Treatment is aimed at reversal of muscarinic signs with atropine and enzyme reactivation by pralidoximes. Frequent atropine doses or continuous titrated infusions are used to achieve drying of secretions and the resolution of bradycardia [11,12]. Tachycardia, however, is not a contraindication to atropine administration [12]. The pupillary response (resolution of miosis) is not considered an end point of atropine therapy, as miosis may persist for weeks after significant exposure [11]. In our cases, the miosis was resolved within 12 and 24 h in the girl and boy, respectively.

Unfortunately, atropinization does not reverse either the central or nicotinic cholinergic signs or symptoms, particularly the muscle weakness and/or paralysis. A different dose of pralidoxime or a continuous infusion is used in severe poisoning up to the resolution of the symptoms or restoration of normal plasma PCE levels [13].

This antidote is best used as early as is reasonable before irreversible inhibition of acetylcholinesterase occurs. A loading dose of 25 to 50 mg/kg followed by a repetitive administration or a continuous infusion of 10 to 20 mg/kg per hour is administered until muscle weakness and fasciculation resolve [14].

Note that health care personnel can develop OI through either dermal or respiratory exposure, and measures should be taken in order to avoid this. In our cases the health care personnel involved developed headaches, but this situation was quite easily resolved by aeration of the room where the patients were treated. Moreover, we should advise the personnel to wear gloves, masks and glasses when decontaminating the patient's skin and to hermitically seal the patients' clothes in a closed bag [1].

## Conclusion

This report emphasizes that misuse of OC, even in cases of domestic application, may be life threatening. This can cause acute OI even through the skin.

Management of OI consists of airway management; administration of oxygen and fluid, atropine in increasing doses and pralidoxime; as well as decontamination of the patient's skin.

The involved health care personnel should be aware of the potential risk of becoming intoxicated themselves when taking care of contaminated patients.

PCE analysis is an easy indicator of OI and can be used for treatment monitoring.

## Competing interests

The authors declare that they have no competing interests.

## Authors' contributions

WH intervened the patient in the emergency department and drafted the manuscript. SHI was the supervising physician who diagnosed OI and treated the patients and corrected the manuscript. All authors read and approved the final manuscript.
